# Estimating the Population Sizes of Men Who Have Sex With Men in US States and Counties Using Data From the American Community Survey

**DOI:** 10.2196/publichealth.5365

**Published:** 2016-04-21

**Authors:** Jeremy A Grey, Kyle T Bernstein, Patrick S Sullivan, David W Purcell, Harrell W Chesson, Thomas L Gift, Eli S Rosenberg

**Affiliations:** ^1^ Department of Epidemiology Rollins School of Public Health Emory University Atlanta, GA United States; ^2^ Division of STD Prevention National Center for HIV/AIDS, Viral Hepatitis, STD, and TB Prevention Centers for Disease Control and Prevention Atlanta, GA United States; ^3^ Division of HIV/AIDS Prevention National Center for HIV, Viral Hepatitis, STD, and TB Prevention Centers for Disease Control and Prevention Atlanta, GA United States

**Keywords:** sexual behavior, population, men who have sex with men, demography

## Abstract

**Background:**

In the United States, male-to-male sexual transmission accounts for the greatest number of new human immunodeficiency virus (HIV) diagnoses and a substantial number of sexually transmitted infections (STI) annually. However, the prevalence and annual incidence of HIV and other STIs among men who have sex with men (MSM) cannot be estimated in local contexts because demographic data on sexual behavior, particularly same-sex behavior, are not routinely collected by large-scale surveys that allow analysis at state, county, or finer levels, such as the US decennial census or the American Community Survey (ACS). Therefore, techniques for indirectly estimating population sizes of MSM are necessary to supply denominators for rates at various geographic levels.

**Objective:**

Our objectives were to indirectly estimate MSM population sizes at the county level to incorporate recent data estimates and to aggregate county-level estimates to states and core-based statistical areas (CBSAs).

**Methods:**

We used data from the ACS to calculate a weight for each county in the United States based on its relative proportion of households that were headed by a male who lived with a male partner, compared with the overall proportion among counties at the same level of urbanicity (ie, large central metropolitan county, large fringe metropolitan county, medium/small metropolitan county, or nonmetropolitan county). We then used this weight to adjust the urbanicity-stratified percentage of adult men who had sex with a man in the past year, according to estimates derived from the National Health and Nutrition Examination Survey (NHANES), for each county. We multiplied the weighted percentages by the number of adult men in each county to estimate its number of MSM, summing county-level estimates to create state- and CBSA-level estimates. Finally, we scaled our estimated MSM population sizes to a meta-analytic estimate of the percentage of US MSM in the past 5 years (3.9%).

**Results:**

We found that the percentage of MSM among adult men ranged from 1.5% (Wyoming) to 6.0% (Rhode Island) among states. Over one-quarter of MSM in the United States resided in 1 of 13 counties. Among counties with over 300,000 residents, the five highest county-level percentages of MSM were San Francisco County, California at 18.5% (66,586/359,566); New York County, New York at 13.8% (87,556/635,847); Denver County, Colorado at 10.5% (25,465/243,002); Multnomah County, Oregon at 9.9% (28,949/292,450); and Suffolk County, Massachusetts at 9.1% (26,338/289,634). Although California (n=792,750) and Los Angeles County (n=251,521) had the largest MSM populations of states and counties, respectively, the New York City-Newark-Jersey City CBSA had the most MSM of all CBSAs (n=397,399).

**Conclusions:**

We used a new method to generate small-area estimates of MSM populations, incorporating prior work, recent data, and urbanicity-specific parameters. We also used an imputation approach to estimate MSM in rural areas, where same-sex sexual behavior may be underreported. Our approach yielded estimates of MSM population sizes within states, counties, and metropolitan areas in the United States, which provide denominators for calculation of HIV and STI prevalence and incidence at those geographic levels.

## Introduction

In the United States, male-to-male sexual transmission accounted for 58-65% of human immunodeficiency virus (HIV) diagnoses from 2009 to 2013 [1], despite the fact that a relatively small proportion of men in the United States are men who have sex with men (MSM) [2]. Prior work on estimating the population size of MSM in the United States [2-5] and at the city [6] and state [3,7-10] levels show that prevalence and incidence rates of HIV and some sexually transmitted infections (STIs) are higher among MSM than other groups. In order to estimate the prevalence or incidence rates of HIV or other STIs among MSM in additional areas, we need to estimate the denominator of population size [2].

Having male sex partners is not necessarily the same as self-identification as gay, bisexual, or queer*.* MSM defines a group of men behaviorally and temporally, and is preferred by public health researchers over identities such as gay or bisexual men because behavior, not identity, leads to sexual transmission of HIV and STIs. Many MSM self-identify as gay or bisexual, but not all. Thus, reports such as a recent Gallup publication [11] that estimate population sizes of lesbian, gay, bisexual, or transgender (LGBT) individuals have limited use for public health. The choice of timeframe influences the estimated percentage of MSM among adult men, and consequently, the estimated size of the MSM population. Most studies use “sex with a man in the past 12 months,” “sex with a man in the past 5 years,” or “any sex with a man ever,” with longer recall periods leading to higher population size estimates [2].

Data regarding cohabitating same-sex partners are collected by the US Census Bureau, but behavioral data on same-sex behavior among men are not. Therefore, researchers studying MSM populations often use estimates from national probability surveys such as the General Social Survey (GSS) [12], National Health and Social Life Survey (NHSLS) [13], National Health and Nutrition Examination Survey (NHANES) [14], and National Survey of Family Growth (NSFG) [4,5,15]. The most recent effort to synthesize data from multiple studies in order to estimate the percentage of MSM among adult men in the United States comes from a meta-analysis of these and other data sources by Purcell and colleagues [2]. However, given uneven geographic dispersion of MSM in the United States, national estimates are inadequate for state and local prevention planning. Examining HIV prevalence and incidence at smaller geographic levels, and comparing HIV burden among MSM in different areas, requires estimation approaches at finer levels.

Several methods have been proposed to estimate state and local population sizes of MSM. Some researchers begin with HIV prevalence assumptions and work backward to determine the population size of MSM in a given area. For example, Lieb and colleagues [16] used data on HIV prevalence among a probability sample of MSM to estimate the number of MSM in six large metropolitan statistical areas (MSAs) in Florida. Other researchers have used data from the US Census Bureau and from large, national health surveys to generate state [3,7,10] and county [9,17-19] MSM population estimates. Gates and Black [20] reported findings separately from the GSS and NHSLS as well as from the 1990 US Census.

One recent approach used both data from the American Community Survey (ACS) and NHSLS estimates [7]. Lieb and colleagues [10] proposed two models to estimate state population sizes for MSM. The first, Model A, assumes different percentages of MSM among men in urban, suburban, and rural areas. For those percentages, Lieb et al. [10] refer to estimates reported by Laumann and colleagues [13] from the 1992 NHSLS. They multiply these percentages by each state’s proportion of total population in rural, suburban, and urban areas, then multiply the result by the 2007 midyear population estimates from the US Census Bureau [3,10]. For Model B, they weight the overall percentage of MSM among US adult men, estimated to be 6% from the 2002 NSFG [3,5,10], according to the representation of same-sex male (SSM) households in a state, relative to the overall proportion of SSM households in the United States as reported in the 2000 Census. They then multiply these weighted percentages by the population in each state, again taken from the 2007 midyear population estimates. The final state estimates are the mean of Models A and B.

Here, we create a new method to estimate the population sizes of MSM in US states, counties, and core-based statistical areas (CBSAs). Our approach uses elements of Lieb et al.’s [10] Models A and B, data on total and SSM households from ACS 2009 to 2013 [21], urbanicity-stratified estimates of the percentage of adult men who had sex with a man in the past year from NHANES [22], and the meta-analytic estimate of the national percentage of adult men who had sex with a man in the past 5 years [2]. By estimating population sizes at smaller geographic levels and within urbanicity strata, we hope to provide public health practitioners and policy makers with a useful tool for determining disease burden and allocating resources at state and county levels, including among nonurban areas.

## Methods

### Data

We used data from the ACS 5-year summary file, 2009 to 2013, to obtain the total number of households, total number of SSM households (male householder and male partner), and total number of men aged 18 years and older for each county in the United States (Multimedia Appendix 1) [21]. The ACS is a supplement to the decennial census that provides annual updates to housing and demographic statistics for the United States [23]. Approximately 1 in 38 US households are randomly sampled each year, and the selected individuals respond using either Web-based or paper questionnaires. Staff from the US Census Bureau follow up with individuals who do not respond, in order to improve response rates.

ACS data are publicly available as 1-, 3-, or 5-year summary files or as a Public Use Microdata Sample (PUMS), which contains a de-identified and unaggregated sample of ACS data. The 1- and 3-year summary files are limited to areas with populations of 65,000 or 20,000 or more, respectively. However, the 5-year ACS summary files contain data at all available geographic areas. We did not include data from US territories.

To more accurately describe where MSM reside, we supplemented data from the ACS using the urbanicity categories produced by the National Center for Health Statistics (NCHS) [24]. According to the NCHS classification scheme, counties fall into six categories: central (ie, inner city) or fringe (ie, suburban) portions of large MSAs (population size ≥ 1,000,000 population), medium-sized MSAs (population size of 250,000-999,999), small MSAs (population of < 250,000), micropolitan area (counties that contain all or part of a city of 10,000 or more), and noncore (counties that do not contain any part of a city of 10,000 or more) [24]. In order to incorporate urbanicity-specific percentages of MSM among adult men, we then collapsed the categories according to the four-level urbanicity classification used by Oster et al. [22]: large central metropolitan county, large fringe metropolitan county, medium/small metropolitan county, and nonmetropolitan county.

### Analysis

**Figure 1:**
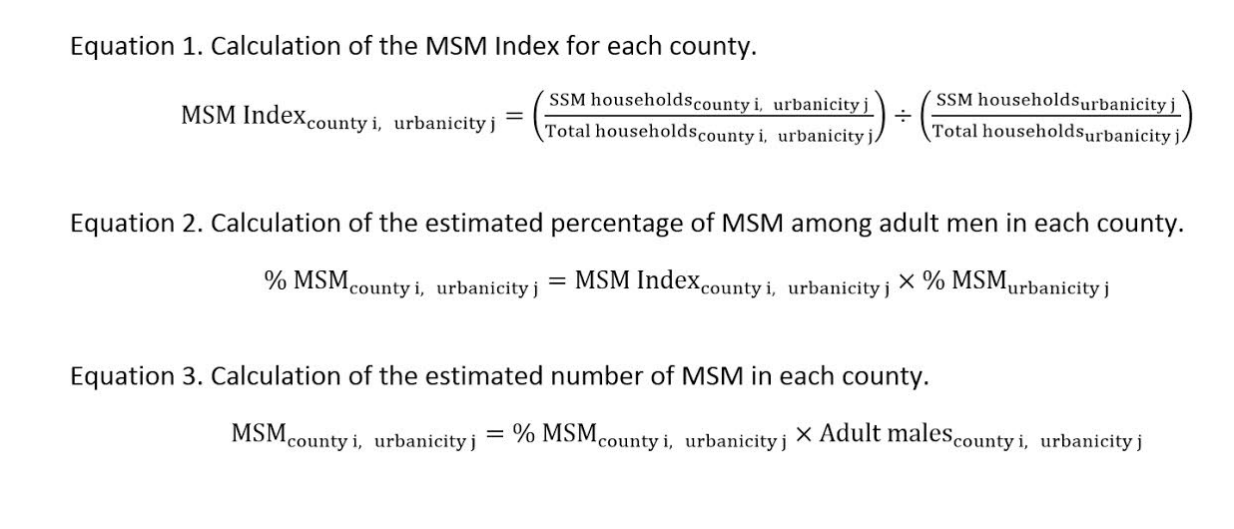
Equations 1–3

We developed a method to estimate small-area MSM populations by combining two models reported by Lieb et al. [10]. The first, Model A, applied estimates of the percentage of MSM among adult men, stratified by urbanicity, to the adult male population. The second, Model B, weighted the national MSM percentage according to the relative representation of SSM households among all households in an area, referred to as the MSM Index. We combined these two models into a single model by stratifying the MSM Index formula to determine the urbanicity-specific relative representation of SSM households (Figure 1, Equation 1). We then multiplied this within-stratum MSM Index to the urbanicity-specific estimated percentage of MSM among adult men from NHANES, as reported by Oster et al. [22] (Figure 1, Equation 2), to arrive at the percentage of males who are MSM in each county. Next, for each county, the number of MSM was estimated by multiplying the MSM percentage by the total adult males (Figure 1, Equation 3).

**Figure 2:**
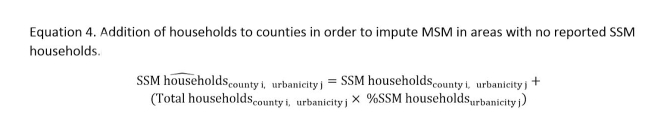
Equation 4.

By calculating the MSM Index within strata of urbanicity, we expected to reduce inter-urbanicity differences in same-sex cohabitation and reporting among MSM due to stigma. However, 35.4% (1112/3143) counties had no reported SSM households, and consequently had MSM Index values and estimated MSM population sizes of zero, which likely reflected these biases in detection of MSM. To impute MSM in these areas for our final estimates, while preserving the relative population sizes based on SSM households, we added households to both the numerator and denominator of the above equations. For each county, we increased the number of SSM households and the number of total households by adding the urbanicity-specific percentage of SSM households (Figure 2, Equation 4).

As an example of our imputation method, we will use two hypothetical nonmetropolitan counties. The total percentage of SSM households among all households in nonmetropolitan counties in our data was approximately 0.1%. For a county with 1000 households, of which zero were SSM households, we added one SSM household, or 0.1% of 1000. This meant that, for the part of our model that calculated urbanicity-specific indices, the new totals for that county were 1001 households, of which one was a SSM household. For another nonmetropolitan county with 20,000 households, of which 15 were SSM households, we added 20 SSM households, for a new total of 20,020 households and 35 SSM households. By adding a proportionate number of SSM households to all counties, we effectively maintained the relative representation of SSM households within urbanicity strata while estimating at least some MSM in counties with no SSM households. Because the index was used as a way of weighting the percentage of MSM among adult men in each county and not as a direct method of estimation, adding SSM households did not add MSM to our final population estimates.

For our analysis, we chose to estimate the number of men who had sex with men within the past 5 years, rather than the past 12 months or over the lifetime, as others have reported [2]. Using past-year estimates might underestimate the total sexually active population, particularly because we are using 5-year population estimates, while lifetime estimates would do the opposite: under that scenario, all men who had sex with another man would be counted, regardless of how recent or frequent the behavior. Because NHANES only has data regarding same-sex sex in the past 12 months and over a lifetime, we scaled our results to sum to 3.9% of the US adult male population, the estimated national percentage of adult men who had sex with a man within the past 5 years from the Purcell et al. meta-analysis [2].

All analyses were conducted using R Studio, version 0.98.953 [25]. Data were analyzed at the county level and aggregated to state and the Office of Management and Budget’s core based statistical areas (CBSAs). CBSAs refer to both metropolitan and micropolitan statistical areas. MSAs are CBSAs with at least 50,000 people. Micropolitan statistical areas have fewer than 50,000 people.

## Results

### State Population

Using Purcell et al.’s [2] estimate of 3.9% MSM (past 5 years) among US adult men and summary data from the 2009 to 2013 ACS, we estimated that there are approximately 4,503,080 MSM in the United States. Table 1 presents the state-level population sizes of MSM, ranked from largest MSM population to least. California, which has 12.1% (13,997,953/115,463,694) of the US adult male population according to ACS estimates from 2009 to 2013, had the largest percentage of the US MSM population at 17.6% (792,750/4,503,080). Furthermore, over one-half of US MSM resided in that and the next six states: Texas at 8.3% of US MSM (371,781/4,503,080; New York at 8.2% of US MSM (371,087/4,503,080); Florida at 7.6% of US MSM (340,163/4,503,080); Illinois at 4.4% of US MSM (199,486/4,503,080); Pennsylvania at 3.6% of US MSM (162,745/4,503,080); and Ohio at 3.2% of US MSM (144,367/4,503,080). Despite 52.9% (2,382,379/4,503,080) of the US MSM population residing in those seven states, they represent only 44.6% (51,508,277/115,463,694) of the US adult male population.

### County Population

**Figure 3:**
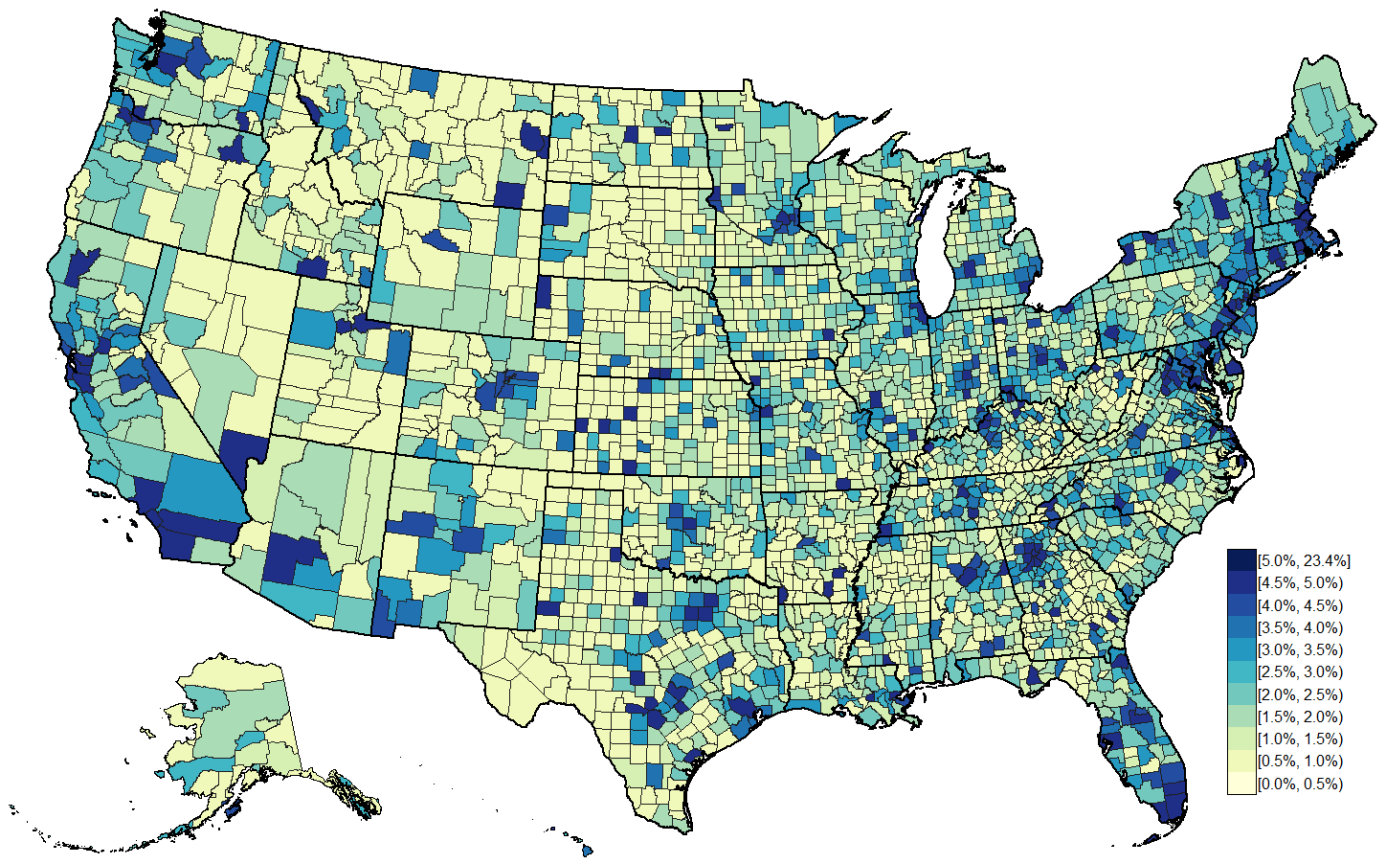
Estimated percentage of adult men who had sex with a man in the past 5 years, using housing and population estimates from the 2009-2013 American Community Survey.

Of the 3143 counties or county-equivalent areas in the United States, we estimated that over one-half of the total US MSM population resided in only 51 (Table 2). The largest number of MSM lived in Los Angeles County, California at 5.6% of US MSM (251,521/4,503,080), followed by cook County, Illinois at 2.8% of US MSM (125,923/4,503,080); Maricopa County, Arizona at 2.0% of US MSM (87,894/4,503,080); New York County, New York at 1.9% of US MSM (87,556/4,503,080); and Harris County, TX at 1.9% of US MSM (83,401/4,503,080). A total of 9.9% (310/3143) US counties had fewer than 20 MSM, and 39.5% (1242/3143) had fewer than 100 (Figure 3).

**Table 1:** Estimated MSM populations in 50 states and the District of Columbia, ranked by size of MSM population, using housing and population estimates from the American Community Survey, 2009-2013.

		Adult males	MSM	US MSM	
Rank	State	N	n (%)	% of total	Cumulative % of MSM
1	California	13,997,953	792,750 (5.7%)	17.6%	17.6%
2	Texas	9,189,027	371,781 (4.0%)	8.3%	25.9%
3	New York	7,247,605	371,087 (5.1%)	8.2%	34.1%
4	Florida	7,283,572	340,163 (4.7%)	7.6%	41.7%
5	Illinois	4,728,089	199,486 (4.2%)	4.4%	46.1%
6	Pennsylvania	4,798,340	162,745 (3.4%)	3.6%	49.7%
7	Ohio	4,263,691	144,367 (3.4%)	3.2%	52.9%
8	New Jersey	3,257,962	132,520 (4.1%)	2.9%	55.8%
9	Georgia	3,522,525	131,374 (3.7%)	2.9%	58.8%
10	Michigan	3,671,762	113,860 (3.1%)	2.5%	61.3%
11	Virginia	3,030,663	112,785 (3.7%)	2.5%	63.8%
12	Washington	2,590,196	111,960 (4.3%)	2.5%	66.3%
13	Massachusetts	2,477,594	111,625 (4.5%)	2.5%	68.8%
14	Arizona	2,393,283	110,344 (4.6%)	2.5%	71.2%
15	North Carolina	3,536,017	103,010 (2.9%)	2.3%	73.5%
16	Maryland	2,136,890	84,465 (4.0%)	1.9%	75.4%
17	Minnesota	2,000,472	83,027 (4.2%)	1.8%	77.2%
18	Tennessee	2,357,860	73,639 (3.1%)	1.6%	78.9%
19	Colorado	1,939,236	73,357 (3.8%)	1.6%	80.5%
20	Missouri	2,219,565	70,783 (3.2%)	1.6%	82.1%
21	Indiana	2,389,263	70,103 (2.9%)	1.6%	83.6%
22	Oregon	1,472,740	61,607 (4.2%)	1.4%	85.0%
23	Wisconsin	2,154,753	59,078 (2.7%)	1.3%	86.3%
24	Nevada	1,038,437	51,726 (5.0%)	1.1%	87.4%
25	Kentucky	1,621,844	47,034 (2.9%)	1.0%	88.5%
26	Connecticut	1,334,105	43,313 (3.2%)	1.0%	89.4%
27	Louisiana	1,665,801	41,492 (2.5%)	0.9%	90.4%
28	Alabama	1,754,583	40,600 (2.3%)	0.9%	91.3%
29	Oklahoma	1,394,881	37,739 (2.7%)	0.8%	92.1%
30	District of Columbia	239,916	36,775 (15.3%)	0.8%	92.9%
31	South Carolina	1,726,807	36,316 (2.1%)	0.8%	93.7%
32	Utah	962,285	33,294 (3.5%)	0.7%	94.5%
33	Rhode Island	395,905	23,815 (6.0%)	0.5%	95.0%
34	Kansas	1,054,271	22,900 (2.2%)	0.5%	95.5%
35	Iowa	1,145,708	20,753 (1.8%)	0.5%	96.0%
36	Arkansas	1,076,736	19,264 (1.8%)	0.4%	96.4%
37	Mississippi	1,063,557	18,992 (1.8%)	0.4%	96.8%
38	New Mexico	762,051	17,969 (2.4%)	0.4%	97.2%
39	Hawaii	534,961	15,411 (2.9%)	0.3%	97.6%
40	Maine	511,631	15,071 (2.9%)	0.3%	97.9%
41	New Hampshire	507,277	14,122 (2.8%)	0.3%	98.2%
42	Nebraska	678,518	13,199 (1.9%)	0.3%	98.5%
43	West Virginia	716,528	13,063 (1.8%)	0.3%	98.8%
44	Delaware	335,554	13,049 (3.9%)	0.3%	99.1%
45	Idaho	574,213	9,907 (1.7%)	0.2%	99.3%
46	Vermont	243,332	7,069 (2.9%)	0.2%	99.5%
47	Montana	386,653	6,374 (1.6%)	0.1%	99.6%
48	South Dakota	309,108	5,171 (1.7%)	0.1%	99.7%
49	Alaska	278,464	5,074 (1.8%)	0.1%	99.8%
50	North Dakota	270,992	4,447 (1.6%)	0.1%	99.9%
51	Wyoming	220,518	3,225 (1.5%)	0.1%	100.0%
	Total	115,463,694	4,503,080 (3.9%)		

**Table 2:** The 51 US counties with the largest estimated MSM populations, representing approximately one-half of the estimated US MSM population and ranked according to size of MSM population, using housing and population estimates from the American Community Survey, 2009-2013.

		Adult males	MSM	US MSM	
Rank	County	State	N	n (%)	% of total	Cumulative % of MSM
1	Los Angeles County	CA	3,666,190	251,521 (6.9%)	5.6%	5.6%
2	Cook County	IL	1,905,622	125,923 (6.6%)	2.8%	8.4%
3	Maricopa County	AZ	1,408,797	87,894 (6.2%)	2.0%	10.3%
4	New York County	NY	635,847	87,556 (13.8%)	1.9%	12.3%
5	Harris County	TX	1,490,581	83,401 (5.6%)	1.9%	14.1%
6	San Diego County	CA	1,204,728	80,968 (6.7%)	1.8%	15.9%
7	Riverside County	CA	794,695	70,803 (8.9%)	1.6%	17.5%
8	San Francisco County	CA	359,566	66,586 (18.5%)	1.5%	19.0%
9	Dallas County	TX	855,958	64,385 (7.5%)	1.4%	20.4%
10	Orange County	CA	1,134,443	62,190 (5.5%)	1.4%	21.8%
11	King County	WA	769,969	61,752 (8.0%)	1.4%	23.2%
12	Kings County	NY	895,148	59,767 (6.7%)	1.3%	24.5%
13	Miami-Dade County	FL	956,927	59,733 (6.2%)	1.3%	25.8%
14	Broward County	FL	664,314	58,629 (8.8%)	1.3%	27.1%
15	Clark County	NV	744,929	46,529 (6.2%)	1.0%	28.2%
16	Queens County	NY	855,853	45,656 (5.3%)	1.0%	29.2%
17	Alameda County	CA	578,149	40,924 (7.1%)	0.9%	30.1%
18	Hennepin County	MN	441,369	37,611 (8.5%)	0.8%	30.9%
19	Santa Clara County	CA	689,137	37,041 (5.4%)	0.8%	31.7%
20	District of Columbia	DC	239,916	36,775 (15.3%)	0.8%	32.5%
21	Sacramento County	CA	517,617	34,556 (6.7%)	0.8%	33.3%
22	Tarrant County	TX	645,094	34,529 (5.4%)	0.8%	34.1%
23	Philadelphia County	PA	550,353	33,549 (6.1%)	0.7%	34.8%
24	Bexar County	TX	621,564	32,401 (5.2%)	0.7%	35.5%
25	Franklin County	OH	431,661	31,220 (7.2%)	0.7%	36.2%
26	Travis County	TX	407,740	30,741 (7.5%)	0.7%	36.9%
27	Orange County	FL	438,963	30,732 (7.0%)	0.7%	37.6%
28	Fulton County	GA	348,541	30,169 (8.7%)	0.7%	38.3%
29	Wayne County	MI	638,235	30,161 (4.7%)	0.7%	38.9%
30	Multnomah County	OR	292,450	28,949 (9.9%)	0.6%	39.6%
31	Hillsborough County	FL	461,567	28,246 (6.1%)	0.6%	40.2%
32	Middlesex County	MA	577,698	28,122 (4.9%	0.6%	40.8%
33	Allegheny County	PA	466,388	26,666 (5.7%)	0.6%	41.4%
34	Suffolk County	MA	289,634	26,338 (9.1%)	0.6%	42.0%
35	Cuyahoga County	OH	460,353	25,837 (5.6%)	0.6%	42.6%
36	Denver County	CO	243,002	25,465 (10.5%)	0.6%	43.2%
37	Pinellas County	FL	358,997	25,204 (7.0%)	0.6%	43.7%
38	Suffolk County	NY	556,340	24,597 (4.4%)	0.5%	44.3%
39	San Bernardino County	CA	722,111	24,060 (3.3%)	0.5%	44.8%
40	Salt Lake County	UT	372,182	23,244 (6.2%)	0.5%	45.3%
41	Palm Beach County	FL	510,352	22,727 (4.5%)	0.5%	45.8%
42	Bronx County	NY	469,573	22,370 (4.8%)	0.5%	46.3%
43	Mecklenburg County	NC	336,345	20,920 (6.2%)	0.5%	46.8%
44	DeKalb County	GA	249,589	20,302 (8.1%)	0.5%	47.2%
45	Marion County	IN	323,768	19,553 (6.0%)	0.4%	47.7%
46	Wake County	NC	331,066	19,021 (5.7%)	0.4%	48.1%
47	Contra Costa County	CA	387,213	18,974 (4.9%)	0.4%	48.5%
48	Erie County	NY	344,098	18,706 (5.4%)	0.4%	48.9%
49	Hudson County	NJ	251,902	18,523 (7.4%)	0.4%	49.3%
50	Milwaukee County	WI	339,381	18,428 (5.4%)	0.4%	49.7%
51	Shelby County	TN	321,669	17,466 (5.4%)	0.4%	50.1%

### Core-Based Statistical Areas

By aggregating our county-level findings to CBSAs, we found that 97.4% (4,384,172/4,503,080) of the MSM in our model resided in the 917 CBSAs in the United States. One-half (2,251,068/4,503,080) lived in one of 16 CBSAs (Table 3), all of which were MSAs. Of those residing in a CBSA, the largest population of MSM was in the New York-Newark-Jersey City CBSA at 8.8% of US MSM (397,399/4,503,080); followed by Los Angeles-Long Beach-Anaheim at 7.0% of US MSM (313,711/4,503,080); Chicago-Naperville-Elgin at 3.9% of US MSM (175,118/4,503,080); San Francisco-Oakland-Hayward at 3.2% of US MSM (145,972/4,503,080); and Miami-Fort Lauderdale-West Palm Beach at 3.1% of US MSM (141,088/4,503,080). Thus, 26.1% (1,173,288/4,503,080) of the MSM in the United States live in these five areas.

**Table 3:** The 16 CBSAs with the largest estimated MSM populations, representing one-half of the US MSM population and ranked according to size of MSM population, using housing and population estimates from the American Community Survey, 2009-2013.

		Adult males	MSM	US MSM	
Rank	State	N	n (%)	% of total	Cumulative % of MSM
1	New York-Newark-Jersey City	7,239,158	397,399 (5.5%)	8.8%	8.8%
2	Los Angeles-Long Beach-Anaheim	4,800,633	313,711 (6.5%)	7.0%	15.8%
3	Chicago-Naperville-Elgin	3,443,489	175,118 (5.1%)	3.9%	19.7%
4	San Francisco-Oakland-Hayward	1,700,219	145,972 (8.6%)	3.2%	22.9%
5	Miami-Fort Lauderdale-West Palm Beach	2,131,593	141,088 (6.6%)	3.1%	26.1%
6	Dallas-Fort Worth-Arlington	2,320,338	133,944 (5.8%)	3.0%	29.0%
7	Washington-Arlington-Alexandria	2,113,258	122,895 (5.8%)	2.7%	31.8%
8	Houston-The Woodlands-Sugar Land	2,159,519	103,722 (4.8%)	2.3%	34.1%
9	Atlanta-Sandy Springs-Roswell	1,899,899	102,642 (5.4%)	2.3%	36.3%
10	Philadelphia-Camden-Wilmington	2,189,761	100,293 (4.6%)	2.2%	38.6%
11	Riverside-San Bernardino-Ontario	1,516,806	94,863 (6.3%)	2.1%	40.7%
12	Phoenix-Mesa-Scottsdale	1,557,094	92,825 (6.0%)	2.1%	42.7%
13	Boston-Cambridge-Newton	1,729,903	92,527 (5.3%)	2.1%	44.8%
14	Seattle-Tacoma-Bellevue	1,342,052	82,002 (6.1%)	1.8%	46.6%
15	San Diego-Carlsbad	1,204,728	80,968 (6.7%)	1.8%	48.4%
16	Minneapolis-St. Paul-Bloomington	1,247,688	71,099 (5.7%)	1.6%	50.0%

## Discussion

### Principal Results

We used recent estimates of the population size of US MSM [2], data from ACS 2009 to 2015 [21], and recent estimated percentages of MSM among adult men by urbanicity [22] with an existing estimation method [10] to estimate state-, county-, and CBSA-level populations of MSM. Based on the relative representation of SSM households and prior estimates of MSM percentages in large central metropolitan areas, large fringe metropolitan areas, medium and small metropolitan areas, and nonmetropolitan areas [22], we found that a majority of MSM live within relatively few counties and CBSAs. Our method is a relatively simple, robust approach to estimating small-area population sizes for MSM that can easily be updated as new data become available.

Our findings are consistent with other studies, although ours is the first to use this method at this fine of a geographic level for the entire country. For example, Gallup’s March, 2015 [11] report on the LGBT population sizes found that the San Francisco-Oakland-Hayward, California, metropolitan area had the highest percentage of LGBT individuals among the general population. This same metropolitan area had the highest percentage of MSM among adult men, according to our method. Furthermore, our estimated number of MSM in San Francisco County, 66,586, was very close to a method that incorporated HIV prevalence estimates and HIV diagnoses: Raymond and colleagues [6] estimated 66,487 MSM in the same area in 2010, which is within the timeframe of our ACS data.

Despite similarities with other studies, our results were different from other recent publications, notably the ones from which we derived part of our method. We estimated fewer MSM at the state level than Lieb and colleagues [3] did in their 2011 article. We also estimated fewer MSM at the state and county level in Texas than Campagna et al. [17,18]. However, Lieb et al. [3] and Campagna et al. [17,18] used a higher estimated proportion of MSM in urban areas for their studies [13]. There are also several additional assumptions about geography in their models that likely contribute to differences: Lieb and colleagues [10] use “urbanized,” “within urban cluster,” and “rural” designations from the US Census Bureau to approximate urban, suburban, and rural, as reported by Laumann et al. [13]. However, “urbanized” and “within urban cluster” refer to metropolitan and micropolitan areas, respectively, or areas with more than 50,000 individuals and areas with greater than 2500 individuals but less than 50,000 individuals. Under these definitions, many suburban areas would be considered “urbanized,” while rural communities might be considered “within urban cluster.”

In addition to the different definitions of urbanicity, the percentages cited by Lieb et al. [10], 1% for rural areas, 4% for suburban areas, and 9% for urban areas, represent Laumann and colleagues’ [13] estimates for gay identity, rather than same-sex behavior. Within the identity category, those percentages were derived from individuals in the urban cores and suburbs of the “top 12 urban areas.” Thus, Model A appears to apply an identity-based measure from major metropolitan areas to different classifications from the US Census Bureau.

Our findings substituted the Laumann et al. [13] estimates with those from Oster and colleagues [22]. We also allowed MSM to exist in areas with no reported SSM households, and we scaled our findings to Purcell and colleagues’ [2] national estimate for the percentage of adult men who had sex with a man in the past 5 years. Consequently, our methods and its results represent a new, improved approach to the important work by Lieb and colleagues [3,7-10,19].

### Limitations

We made several assumptions and adjustments to prior methods that may limit the interpretation and use of our results. First, we decided that computing the MSM Index according to stratum would more accurately compare geographic areas, given possible within-urbanicity tendencies for MSM either not to cohabitate or to underreport SSM households. However, it may be that it is more accurate to compare all geographic areas, rather than to generate urbanicity-specific MSM Index values. Second, we used urbanicity-specific MSM percentages from Oster and colleagues [22], rather than the original estimates from Laumann et al. [13]. However, the urbanicity estimates from Laumann et al. [13] are identity-based, and the Oster et al. [22] estimates provided the most congruent urbanicity classifications for Model A. Finally, in order to avoid underestimating the number MSM outside of large urban areas, we imputed a proportional number of MSM to areas with no reported SSM households. It may be that some areas with no SSM households truly also have no MSM. However, the relative percentages of MSM (and resulting MSM population sizes) in all areas was mostly preserved because we altered the number of households and not the number of individuals, which was used only for weighting.

In addition to our method, our findings may be limited by our use of ACS data. The ACS is a sample of the population that is weighted, unlike the decennial census, which contains more data. As a result, inferences based on the ACS may be less accurate than data from the decennial census. ACS might also miss some of the same-sex households that are not in urban areas, particularly if they are less likely to respond to a survey other than the decennial census. It could also be due to more cohabitation, including marriage, among same-sex couples due to differences in legislation permitting marriage. However, because our data span several years, we cannot determine the extent to which policies and laws regarding marriage influence geographic differences.

### Conclusions

Small-area estimates of MSM populations that incorporate the most recent data and estimates available may provide a useful tool to public health practitioners and policy makers for determining the burden of HIV and STIs among MSM in local contexts and planning prevention and treatment responses. Our method produced similar results to a recent effort to estimate MSM population sizes in San Francisco County but different from other studies that used a similar method, largely due to differences in the assumptions underlying the models. The method we presented can be updated annually as new ACS data are released, which would provide counties and larger geographic areas with up-to-date population sizes and, potentially, incidence and prevalence rates. These local statistics would allow for better resource allocation, intervention development, and service delivery. For data from the current analysis and for future updates, visit the study website [26].

The findings and conclusions in this manuscript are solely the responsibility of the authors and do not necessarily represent the official views of the Centers for Disease Control and Prevention or the Department of Health and Human Services.

## Appendix

Multimedia Appendix 1 Variable names and locations of key data from the American Community Survey, 2009-2013.

## Abbreviations

ACS: American Community Survey
CBSA: core-based statistical area
GSS: General Social Survey
HIV: human immunodeficiency virus
MSA: metropolitan statistical area
MSM: men who have sex with men
NHANES: National Health and Nutrition Examination Survey
NHSLS: National Health and Social Life Survey
NSFG: National Survey of Family Growth
SD: standard deviation
SSM: same-sex male
STI: sexually transmitted infection
