# Supplementary material for: Estimating the Population Sizes of Men Who Have Sex With Men in US States and Counties Using Data From the American Community Survey
Source: JMIR Public Health Surveill. 2016 Apr 21;2(1):e14. doi: 10.2196/publichealth.5365 (PMC4873305; doi:10.2196/publichealth.5365)
Supplement: Multimedia Appendix 1 [file publichealth_v2i1e14_app1.pdf]

Variable	Table ID	Sequence	Line
Total households	B110099	37	1
Same-sex male households	B110099	37	3
Men aged 18 years and older	B05003A	9	8
